# Which Reference Should We Use for EEG and ERP practice?

**DOI:** 10.1007/s10548-019-00707-x

**Published:** 2019-04-29

**Authors:** Dezhong Yao, Yun Qin, Shiang Hu, Li Dong, Maria L. Bringas Vega, Pedro A. Valdés Sosa

**Affiliations:** 1 The Clinical Hospital of Chengdu Brain Science Institute, MOE Key Lab for NeuroInformation, University of Electronic Science and Technology of China, No. 2006, Xiyuan Ave., West Hi-Tech Zone, Chengdu, 611731 China; 2 School of Life Science and Technology, Center for Information in Medicine, University of Electronic Science and Technology of China, Chengdu, China; 3 Sichuan Institute for Brain Science and Brain-Inspired Intelligence, Chengdu, China

**Keywords:** REST reference, Average reference, Linked-mastoids reference, Laplacian, Bipolar reference

## Abstract

Which reference is appropriate for the scalp ERP and EEG studies? This unsettled problem still inspires unceasing debate. The ideal reference should be the one with zero or constant potential but unfortunately it is well known that no point on the body fulfills this condition. Consequently, more than ten references are used in the present EEG-ERP studies. This diversity seriously undermines the reproducibility and comparability of results across laboratories. A comprehensive review accompanied by a brief communication with rigorous derivations and notable properties (Hu et al. Brain Topogr, 2019. 10.1007/s10548-019-00706-y) is thus necessary to provide application-oriented principled recommendations. In this paper current popular references are classified into two categories: (1) unipolar references that construct a neutral reference, including both online unipolar references and offline re-references. Examples of unipolar references are the reference electrode standardization technique (REST), average reference (AR), and linked-mastoids/ears reference (LM); (2) non-unipolar references that include the bipolar reference and the Laplacian reference. We show that each reference is derived with a different assumption and serves different aims. We also note from (Hu et al. 2019) that there is a general form for the reference problem, the ‘no memory’ property of the unipolar references, and a unified estimator for the potentials at infinity termed as the regularized REST (rREST) which has more advantageous statistical evidence than AR. A thorough discussion of the advantages and limitations of references is provided with recommendations in the hope to clarify the role of each reference in the ERP and EEG practice.

## Background

**Fig. 1:**
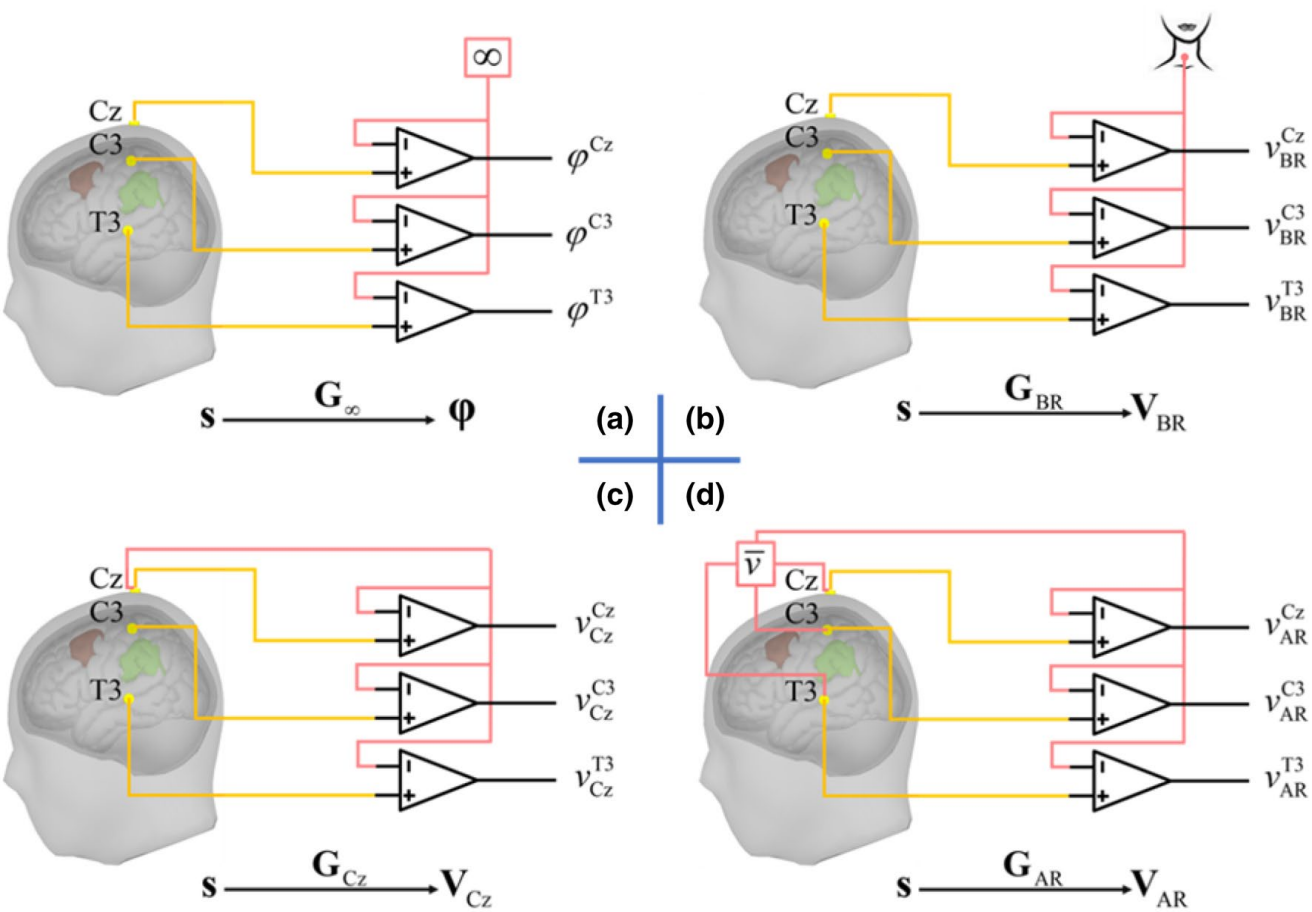
Schematic representation of unipolar references. **a** Infinity/Zero Reference; **b** Recording reference electrode at neck; **c** Recording reference electrode Cz; **d** Average reference. (Reproduced from Hu et al. 2018c)

Since the first report of the human EEG in 1929 (Berger 1929; Gloor 1969), the reference issue has long been debated and Berger had called attention to the reference issue at the very beginning. The discussion arises from the fact that online recorded signal by one channel is the potential differences of two electrodes, leaving the choice of reference electrode undetermined. Instructively, from 1929 to 1938, Berger examined in 14 papers the types of electrodes, recording sites, and both bipolar vs. unipolar referential recordings (Vaque La 1999). Thus, unipolar and bipolar recordings were almost simultaneously introduced from the discovery of human EEG. The schematic representation of a few unipolar references is displayed in the Fig. 1.

Early, there are misleading examples of poor references. A case in point is the ever popular linked-mastoids/ears (LM) reference. In 1930s, EEG recordings with LM were used by Gibbs and Lennox to study grand mal and psychomotor (partial complex) seizures (Gibbs et al. 1936). This work spurred international interest in the role of EEG in clinical epilepsy and firmly linked the term “psychomotor epilepsy” to a specific EEG pattern. However, the authors failed to accurately localize the origin of psychomotor seizures as the LM distorted the field maps (Faux et al. 1990; Feindel et al. 2009; Stone and Hughes 2013). Subsequently, it was shown that LM seriously biases EEG power (Niedermeyer 1987) and coherence spectra (Fein et al. 1988), confounding the interpretation of results (Shaw 1984; Travis 1994). Furthermore, this reference near the neck tends to pick up electromyography (EMG) and electrocardiogram (EKG) artifacts (Luck 2014). It illustrates that the intuitively appealing reference may be fraught with difficulties. The problem is not limited to the LM. Many other body sites have been explored, such as the angle of the jaw, the chin, the tip of the nose, and the neck, etc. These attempts were similarly problematic due to the contamination from EMG and EKG and the difficulty of interpreting the field maps.

In 1940s, a better reference was inspired by the EKG technology. The Wilson EKG common terminal reference sought for a zero-potential reference by combining leads from three limbs. This suggested the average reference (AR) by connecting all EEG electrodes through high resistances in order and then taking the common junction as a reference. In 1950, the first clinical use of AR was reported (Goldman 1950), and it stated that “*if the EEG sources consist of a large number of randomly placed and randomly oriented dipoles, a rather constantly zero average will be obtained over the surface of the scalp. Experience with the average monopolar reference electrode shows that this is usually approached in practice*” (Offner 1950). The AR is currently one of the most widely adopted references. And it is now implemented by offline re-referencing instead of the original online recording setup. The assumption of ‘sum to zero’ behind AR was partly buttressed by the demonstration that the surface potential integral of a dipole in a layered spherical surface is zero (Bertrand et al. 1985). Hereafter, this theoretical result has been thought true for actual human head as “*it is important to note that the dipolar nature of ERP components means that every component is actually positive over some parts of the head and negative over other parts, summing to zero over the entirety of the head*” (Luck 2014). Given this belief, the AR was advocated as the best reference option. Nunez stated “*when used with large numbers of electrodes…, it often performs reasonably well*” (Nunez and Srinivasan 2006), and “*AR errors are due to (1) limited electrode density and (2) incomplete electrode coverage (sampling only the upper part of head). If these errors were fully eliminated (only possible in detached heads), the AR would provide the desired gold standard, that is, the nominal reference with respect to infinity*” (Nunez 2010). Alas, this sweet dream has recently been shown to be just a dream. The surface integral of EEG potentials is not zero for a homogenous and isotropic realistic head geometry shape that deviates from a sphere (Yao 2017). Thus, AR as an approximation to zero potential is subject to many conditions and not universally valid.

In 2001, the reference electrode standardization technique (REST) was proposed to approximately reconstruct EEG potentials with infinity reference (Yao 2001). REST utilizes that EEG recordings are the activities generated by neural current sources but attenuated and mixed by the volume conductor. These currents are independent of any reference. Thus, the neural current sources are taken as the bridge to transform one reference recordings to another. It is utilized offline to transfer a nonzero reference recording to a recording with the approximated zero reference. Besides, REST has been extensively evaluated with various simulations (Zhai and Yao 2004a; Marzetti et al. 2007; Qin et al. 2010; Liu et al. 2015; Qin et al. 2017; Chella et al. 2017; Huang et al. 2018).

Recently, the advantages of REST have been underscored by the demonstration that both REST and AR are particular cases of a unified reference estimator under the Bayesian statistical framework (Hu et al. 2018c); the difference is that the prior probability for REST is based on the physics of volume conduction whereas that of AR relies on the statistical assumption that multichannel EEG recording are uncorrelated. This has consequences for the relative performance of each method as we shall see later. Before proceeding we will emphasize that while the reference choice is an essential problem it is not a magical solution. For example, mixing effects of volume conduction are still present in the signal recorded with respect to infinity. However, as we shall elaborate in the following, use of the correct reference solids the basis for subsequent de-mixing.

An approach that sidesteps the unipolar reference is to avoid studying electric potential altogether but rather calculate the current source density (CSD) (Hjorth 1975), an estimate of current flow through the scalp surface (Yao 2002a). CSD is independent of the reference and helps localizing brain activity close to the electrodes. However, as a Laplacian spatial filterer, CSD is more sensitive to noise with broadband spatial spectra than to physiological sources, and it probes local shallow neural activities at the expense of widely distributed deep source activities. Nevertheless, none of current algorithms can overcome these challenges.

Additionally, the bipolar recordings widely used in the clinical practice are free of the unipolar reference. Bipolar recordings are the potential differences of two nearby electrodes, canceling the effect of unipolar reference. It is proportional to the local current through these two electrodes.

As summarized in (Luck 2014): “*the reference is an absolutely fundamental aspect of EEG/ERP recordings. If you don’t fully understand referencing, you won’t understand the signal that you are recording*”. Everyone agrees on that the lack of consensus in reference choice causes considerable confusion. It is hence necessary to timely review recent progresses in this field (Yao 2017; Hu et al. 2018b, c; 2019) and integrate them with previous work (Yao 2001; Zhai and Yao 2004a). This would complement to the existing reviews, such as (Nunez and Srinivasan 2006; Nunez 2010). We will attempt tracing the proposal of each reference to its physical basis, examining its mathematical properties, and evaluating its performance with experimental data. This allows to suggest guidelines for the choice of references under specific circumstance.

The exposition includes the appropriate derivations for each concept, but these can be skipped to an intuitive description for the benefit of those not mathematically oriented. Institutive explanations will be marked by the words: *Essential concept*.

While preparing this review, Hu et al. realized that the derivations of AR and REST from the maximum likelihood estimate and the properties of unipolar references haven’t been previously explicitly published. We accompany this paper by the brief communication (Hu et al. 2019).

## The EEG/ERP Reference Problem

### No Constant Point on the Scalp Surface

Assuming a head with several compartments, each with homogeneous and isotropic conductivity, then Poisson’s equation is valid for EEG and ERP potentials (Gulrajani 1998):

1 $$\nabla^{2} {\varvec\upvarphi }(\vec{r}) = - {\mathbf{s}}(\vec{r})$$

where **φ** is the potential recorded by the EEG/ERP electrode placed on the scalp; **s** is an equivalent current source density (Plonsey and Heppner 1967; Malmivuo and Plonsey 1995; Yao and He 1998), located at anywhere in the brain that generates the potential **φ**. Combining (1) with the boundary conditions over the scalp surface and internal interfaces of compartments, the scalp EEG/ERP may be linked to the neural source currents inside the brain, laying the basis for the EEG/ERP forward and inverse problems (Yao and He 2003). Based on (1) and head model with boundary conditions, the physiological potential **φ** with the infinity reference related to the neural sources **s** is

2 $${\varvec{\upvarphi}}={\mathbf{G}}_{\infty } {\mathbf{s}}$$

Here **G**_∞_ known as the lead field matrix, expresses the forward model theoretically computed with the infinity reference.

*Essential concept* Even when measured potentials with infinity reference, activities of the neural sources **s** are attenuated and mixed by the properties of different head compartments, e.g. scalp skin, skull, brain etc. This effect is summarized in the lead field.

**Fig. 2:**
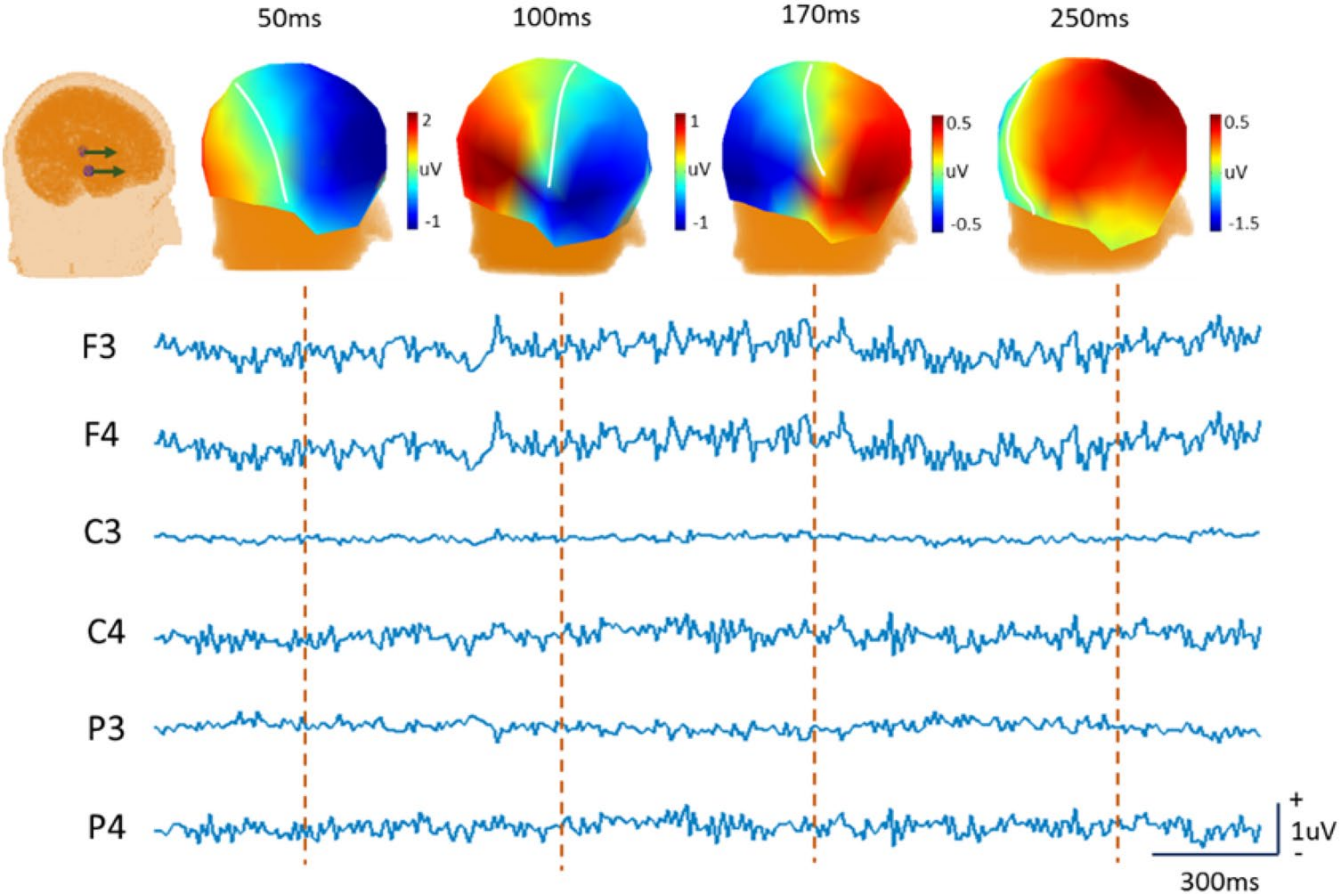
Simulated data shows the inconsistency of zero potential line. The heads show the position of two dipoles and the topographic maps at four time-samples, where the white curve over the scalp is the zero-potential curve which is dynamical momently. The traces show the EEG temporal processes over six electrodes

For a homogeneous spherical head model, the lead field and the surface potential can be calculated analytically with the closed solution (Yao 2000a). For a realistic head model, the corresponding lead fields can be calculated by the boundary element method (BEM) (Zhai and Yao 2004a), the finite element method (FEM) (Yan et al. 1991), and other discrete methods (Gulrajani 1998). For a dipolar source **s**, the scalp potential is positive over one part of the head and negative over the other, and the potentials at the boundary are zero. In Fig. 2, the simulated EEG was generated by two dynamical sources inside a realistic head model. The head model was built using FEM with the SimBio pipeline (Vorwerk et al. 2018), and the conductivities of gray matter, skull and scalp were set as 0.33, 0.01, and 0.43, respectively. The electrode set is the EGI GSN-HydroCel-129.sfp. The source space is totally consisted of 3471*3 dipoles, oriented to x-, y-, and z- axes. Two dipoles at right and left hemisphere with different time series but fixed to x-axis, were taken as the active neural sources to produce the scalp EEG without considering the recording noise. The topographic maps of EEG/ERP at different instants were displayed. However, due to the time-varying nature of the neural electric sources, this zero-boundary curve is never static, and one cannot practically use it as a reference, that is, there is no a point with time-invariant ‘zero’ potential on the head.

In cognitive ERP domain, this physical phenomena is whimsically termed as “no-Switzerland principle” (Luck 2014): there is no electrically neutral site on the head or body surface. It is underscored that any online reference deviates from the infinity zero reference. Therefore, any waveform recorded with an online unipolar reference is the potential difference between one active electrode and a reference site. Both records the same neural source activities via volume conduction. It is the neural current distribution in the whole brain that produces EEG activities at all electrodes including the reference electrode.

### General Form of the Reference Problem

In practice one can never observe ${\mathbf{\varphi }} \in R^{{N_{c} \times 1}}$, what one observed instead is the referenced data ${\mathbf{v}}_{r} \in R^{{N_{c} \times 1}}$. That is the linear transformation via pre-multiplying the reference operator ${\mathbf{T}}_{r} \in R^{{N_{c} \times N_{c} }}$ with the clean physiological potentials **φ** adding the sensor noise **ε**. The general form of the reference problem is modeled as:

3 $${\mathbf{v}}_{r} = {\mathbf{T}}_{r} {\varvec{\upvarphi }} + {\varvec{\upvarepsilon}}_{r}$$

where **T**_r_ is a non-stochastic matrix of observations, **φ** are potentials with the infinity reference, supposed to be a deterministic, fixed but unknown vector, **ε** are non-observable random sensor noise disturbances (Hu et al. 2019). The EEG reference problem in (3) is apparently an underdetermined linear regression problem.

Without loss of generality, **v**_*r*_ and **ε** are considered to have the multivariate normal distribution. If the sensor noise has an independent identical distribution (IID) across channels, the covariance of the sensor noise in the referenced data will be $\varSigma_{{{\varvec{\upvarepsilon}}_{r} {\varvec{\upvarepsilon}}_{r} }} = \sigma^{2} {\mathbf{T}}_{r} {\mathbf{T}}_{r}^{{\mathbf{T}}}$, because referencing effect is taken on the noise as well during recording (Pascual-Marqui et al. 1994; Hu et al. 2018c).

In this study, **T**_*r*_ of unipolar references is the overwhelming body of the EEG reference issue, as its goal is to approach the ideal potential with infinity reference. Besides, **T**_*r*_ can also be the 1st derivative in the bipolar recordings, which is proportional to the local current density between two adjacent electrodes and the 2nd differential operator in the scalp Laplacian, a possible approximation to the current source density. The latter two are different physical quantifications from EEG potentials.

Two approaches produce the identical results that AR and REST are the estimators of the ideal potentials **φ** with infinity reference (Hu et al. 2019). One approach is to derive AR and REST from the maximum likelihood estimate (MLE) with the linear constraint and a quadratic constraint respectively. An alternative and more flexible approach follows from the Bayesian framework. Since the Bayesian ones are more general and identical to the MLE estimators, we will refer the readers to (Hu et al. 2019) for the details of the MLE estimators. In (Hu et al. 2018c), it was demonstrated that any estimator of the potentials referenced to infinity is the maximum a posterior (MAP) estimator

4 $${\hat{\mathbf{\varphi }}}_{r} = {\varvec{\Sigma}}_{{{\mathbf{\varphi \varphi }}}} {\mathbf{T}}_{r}^{{\mathbf{T}}} ({\mathbf{T}}_{r} {\varvec{\Sigma}}_{{{\mathbf{\varphi \varphi }}}} {\mathbf{T}}_{r}^{{\mathbf{T}}} + \sigma^{2} {\mathbf{T}}_{r} {\mathbf{T}}_{r}^{{\mathbf{T}}} )^{ + } {\mathbf{v}}_{r}$$

where + denotes the Moore–Penrose pseudoinverse. The difference between ${\hat{\mathbf{\varphi }}}_{r}$ estimators is the prior covariance ${\varvec{\Sigma}}_{{{\mathbf{\varphi \varphi }}}}$ assumed for the potentials at infinity. This result is surprising since it brings many techniques till now considered totally different under a unique Bayesian statistical framework. If the potentials **φ** with infinity reference are priori IID over all electrodes, the estimator will correspond to the AR whatever the unipolar reference is. By contrast, if the potentials **φ** with infinity reference are generated by the neural sources with IID, the REST estimator is derived. This will be shown in the “Average Reference (AR)” and “Reference Electrode Standardization Technique (REST)”, respectively.

*Essential concept* All references are the linear combination of the ideal recording with infinity reference which turns a linear transformation through the lead field of actual neural source activity. Therefore estimating ${\hat{\mathbf{\varphi }}}_{r}$ is the solution to a linear underdetermined regression (inverse problem). The AR can be derived by constraining the sum over all electrodes to be zero, or in the Bayesian framework assuming prior independence of multichannel EEG recordings; the REST on the other hand assumes potentials generated by a lead field for which a minimum norm constraint may be imposed, or the assumption of independent neural sources is needed in the Bayesian framework (Hu et al. 2018c, 2019).

### Indeterminacy Principle of Scalp EEG

The origin of EEG reference problem is the volume conduction, which leads to an “indeterminacy principle of scalp EEG”. Seen from (2), on the one hand, it is only by volume conduction that we can observe the scalp potentials; on the other hand, volume conduction obstructs precise knowledge of source positions and their time series. From the work on Helmholtz in the 19th century, it is well known that the EEG inverse problem does not admit a unique solution. In fact, any surface potential distribution can be equivalently generated by a closed source layer containing all the true sources, alternatively, by a multipole series expansion at the coordinate origin (Yao 2000b; Yao and He 2003). This makes it generally very difficult to estimate the precise source activities. As a consequence, the waveforms of EEG/ERP are enveloped in the fog of the unknown reference signal (Yao 2001).

It is clear that any solution to the EEG reference problem must be based on volume conduction thus transforming it into a physically spatial inference problem (Yao 2001; Hu et al. 2018c). Obviously, this fundamental principle has been ignored in the previous efforts, that considered the reference problem as a purely inference issue over time. For example, recent procedures consider unrealistic assumption that the reference signal is statistically independent from the true EEG signals. This leads to mathematically tractable solutions based on various blind source separation methods, e.g. temporal independent or principal component analysis. Unfortunately, the indeterminacy principle indicates that the reference signal is generated by the same source as those of the true activity over all channels. Apparently, the temporal evolution of the reference signal should be quite similar as those of nearby channels, breaking the assumptions of temporal independence. Thus, the reference problem is not because of any temporal process but rather due to the spatial volume conduction. An upshot of this is to tackle the reference problem by considering the volume conduction.

## Theory of Unipolar References

As (Hu et al. 2019) noted, “*Unipolar reference is regarded if all electrodes are referenced to a unique physical reference or a unique virtual reference. The physical reference is usually the electrode (e.g. Cz, Fz, Oz and FCz) placed over the scalp or the body surface during online recording setup. The virtual reference is the linearly combined signal of the recordings from all the electrodes, during offline processing after the EEG data acquisition. Typical examples of virtual references are the LM, AR and REST*.”

The reference operator in (3) for unipolar references (Hu et al. 2018b) is commonly as,

5 $${\mathbf{T}}_{r} = {\mathbf{I}}_{{N_{c} }} - {\mathbf{1f}}_{r}^{{\mathbf{T}}}$$

where **1** is a vector of ones; ${\mathbf{I}}_{{N_{c}}}$ is an identity matrix; ${\mathbf{f}}_{r} \in R^{{N_{c} \times 1}}$ consists of the linear combination weights of all the electrodes. The brief communication (Hu et al. 2019) demonstrated that the properties of unipolar references are ‘no memory’, ‘rank deficient by 1’ namely **T**_*r*_ are all full rank deficent by 1 for all the unipolar references, and ‘orthogonal projector centering’.

*Essential concept* The ‘no memory’ property indicates that any two of the unipolar references can be transformed from one to the other and all the unipolar references are independent (Hu et al. 2019).

### Recording Reference (RR)

It is prevalent that the EEG is recorded with respect to a single physical reference electrode, such as Cz, left or right earlobe, and chin etc. For these online recording references, the reference operator (5) is

6 $${\mathbf{T}}_{RR} = {\mathbf{I}}_{{N_{c} }} - {\mathbf{1f}}_{RR}^{\mathbf{T}} , \, {\mathbf{f}}_{RR} = \left[ {0, \ldots 1, \ldots ,0} \right]^{\mathbf{T}}$$

which is a vector of zeros except for a unique entry being 1 at the corresponding index of the reference electrode.

Early, this single site was chosen with the guess that it would be less or not active compared to the other sites that reflects the activity of circumscribed brain areas. A seemingly promising approach is to select the reference as far as possible from electrodes presumed to reflect the activity of sources of interest. For instance, to study the state of the left temporal lobe, the right ear may be taken as the reference. The main problem is that all channels reflect contributions from both the active electrodes as well as the reference site. The no-Switzerland principle—‘no point on the scalp or the body surface with the neutral potential’ makes it a fantasy to separate the EEG activity with infinity reference from that with a body reference.

However, importance of selecting a proper recording reference has been decreased. One can now easily re-reference the digital EEG offline, given the ‘no memory’ property of unipolar references. In current practice, Cz is widely adopted as the online recording reference since it is easy to secure the electrode contact, avoiding additional artifacts injected. Offline digital processing is a feasible way to rescue the recording reference by reconstructing a neutral reference from the observed EEG data. There are several typical attempts such as LM, AR, and REST discussed in the following.

### Linked Mastoids (LM)

LM assumes that the average of the potentials recorded over two mastoids (ears) is close to zero or neutral, where the reference operator in (5) is

7 $${\mathbf{T}}_{LM} = {\mathbf{I}}_{{N_{c} }} - {\mathbf{1f}}_{LM}^{\mathbf{T}} , \, {\mathbf{f}}_{LM} = \left[ {0, \ldots ,0.5, \ldots ,0.5, \ldots 0} \right]^{\mathbf{T}}$$

which is a vector of zeros except for two entries being 0.5 at the corresponding indexes of two mastoids (ears). The studies using LM are usually exploring the data recorded from electrodes at the middle line of the scalp, such as F3, Fz, F4, C3, Cz, C4, P3, Pz, P4, O1, Oz and O2, etc. However, not based on any principle, this heuristic choice resulted into the ambiguities for example in studies of N170 (Luck 2014).

### Average Reference (AR)

One attempt to estimate the potentials **φ** with infinity reference in (3) is by means of the AR. It is justified that for a perfect layered spherical head with neural currents spreading in an isotropic way, the integral of the potentials over the head surface is zero (Bertrand et al. 1985; Yao 2017). Thus, the average potential over all electrodes might tend to zero and then be suitable as the reference signal. In our formulation (5), it is

8 $${\mathbf{T}}_{AR} = {\mathbf{I}}_{{N_{c} }} - {\mathbf{1f}}_{AR}^{{\mathbf{T}}} , \, {\mathbf{f}}_{AR} = {{\mathbf{1}} \mathord{\left/ {\vphantom {{\mathbf{1}} {N_{c} }}} \right. \kern-0pt} {N_{c} }}$$

which is a vector of ${\mathbf{1} \mathord{\left/ {\vphantom {1 {N_{c} }}} \right. \kern-0pt} {N_{c} }}$ for all the electrodes.

Given the ‘rank deficient by 1’ property, the **T**_*r*_ is always singular (Hu et al. 2019). The estimation of **φ** in (3) is thus a generalized linear inverse problem. The minimum Euclidean norm solution is the special case of (4) with the prior ${\varvec{\Sigma}}_{{{\mathbf{\varphi \varphi }}}} = \alpha^{2} {\mathbf{I}}_{{N_{c} }}$ and the assumption σ^2^ tends to zero (Hu et al. 2018c). Thus, the solution finally simplifies to

9 $${\hat{\mathbf{\varphi }}}_{AR} = {\hat{\mathbf{\varphi }}}_{r} = {\mathbf{T}}_{AR} {\mathbf{\varphi }} + {\varvec{\upvarepsilon}}_{AR}$$

by noting the orthogonal projector centering property ${\mathbf{T}}_{r}^{ + } {\mathbf{T}}_{r} = {\mathbf{T}}_{AR}$ (Hu et al. 2019).

This means that the minimum norm solution of (3) with any **T**_*r*_ is same as applying the AR to the potentials with infinity reference. It also confirms that AR can only be applied to the recorded data that was already transformed by the other unipolar references (Hu et al. 2018a).

*Essential concept* The AR is essentially solving a generalized linear inverse problem to estimate the potentials at infinity. With a priori IID covariance across multichannel recordings, the estimator is the minimum norm solution. This conclusion is valid no matter which unipolar reference one starts from and it is equivalent to applying the AR (Hu et al. 2018c, 2019).

### Reference Electrode Standardization Technique (REST)

REST recognized the fact that EEG activities are ultimately generated by the same sources **s** in (2) whatever reference is used. Therefore, the following version of (3) is valid

10 $${\mathbf{v}}_{r} = {\mathbf{G}}_{r} {\mathbf{s}} + {\varvec{\upvarepsilon}}_{r}$$

where ${\mathbf{G}}_{r} = {\mathbf{T}}_{r} {\mathbf{G}}_{\infty }$ is the modified forward model with the same reference as in the EEG data. With the covariance of the equivalent source over time as ${\varvec{\Sigma}}_{{{\mathbf{ss}}}}$, the solution (4) to the equation (10) is expressed as

11 $${\hat{\mathbf{\varphi }}}_{rREST} = {\mathbf{G}}_{\infty } {\varvec{\Sigma}}_{{{\mathbf{ss}}}} {\mathbf{G}}_{r}^{{\mathbf{T}}} ({\mathbf{G}}_{r} {\varvec{\Sigma}}_{{{\mathbf{ss}}}} {\mathbf{G}}_{r}^{{\mathbf{T}}} + \sigma^{2} {\mathbf{T}}_{r} {\mathbf{T}}_{r}^{{\mathbf{T}}} )^{ + } {\mathbf{v}}_{r}$$

This is the regularized version of REST (rREST) (Hu et al. 2018c). If assuming the equivalent source covariance is ${\varvec{\Sigma}}_{{{\mathbf{ss}}}} = \alpha^{2} {\mathbf{I}}_{{N_{s} }}$ and σ^2^ tends to 0, say, the case of noise free data, it turns as the REST transforming

12 $${\hat{\mathbf{\varphi }}}_{REST} = {\mathbf{G}}_{\infty } ({\mathbf{G}}_{r}^{ + } {\mathbf{v}}_{r} ) = ({\mathbf{G}}_{\infty } {\mathbf{G}}_{r}^{ + } ){\mathbf{v}}_{r} = {\mathbf{R}}_{r} {\mathbf{v}}_{r}$$

where ${\mathbf{R}}_{r} = {\mathbf{G}}_{\infty } {\mathbf{G}}_{r}^{ + }$ is the reference standardization matrix depending on **T**_*r*_ and the equivalent source is approximately estimated as ${\hat{\mathbf{s}}} = {\mathbf{G}}_{r}^{ + } {\mathbf{v}}_{r}$ (Yao 2001).

The REST operator is defined as

13 $${\mathbf{T}}_{REST} = \mathbf{G}_{\infty } ({\mathbf{T}}_{r} \mathbf{G}_{\infty } )^{ + } {\mathbf{T}}_{r}$$

in the formula (9) and the Table 1 of (Hu et al. 2018b). It is demonstrated in (Hu et al. 2019) that REST operator is a unipolar reference and admits the ‘no memory’ property with

14 $${\mathbf{T}}_{REST} = {\mathbf{I}}_{{N_{c} }} - {\mathbf{1f}}_{REST}^{\mathbf{T}} , \, {\mathbf{f}}_{REST} = {{{\mathbf{G}}_{\infty }^{ + \mathbf{T}} {\mathbf{G}}_{\infty }^{ + } {\mathbf{1}}} \mathord{\left/ {\vphantom {{{\mathbf{G}}_{\infty }^{ + \mathbf{T}} {\mathbf{G}}_{\infty }^{ + } {\mathbf{1}}} {\left[ {{\mathbf{1}}^{\mathbf{T}} {\mathbf{G}}_{\infty }^{ + \mathbf{T}} {\mathbf{G}}_{\infty }^{ + } 1} \right]}}} \right. \kern-0pt} {\left[ {{\mathbf{1}}^{\mathbf{T}} {\mathbf{G}}_{\infty }^{ + \mathbf{T}} {\mathbf{G}}_{\infty }^{ + } \mathbf{1}} \right]}}$$

**Table 1:** Factors impacting the unipolar reference signal

	Unipolar Reference	Electrode density	Electrode coverage	Head model (shape, inner conductivity)
Online	Cz, Pz, etc.			
Offline	LM			
	REST	√	√	√
	AR	√	√	√

*Essential concept* REST is a unipolar reference with the no memory property; when one assumes that EEG data are generated by brain sources, REST is in theory the optimal for estimating the potentials at infinity; rREST has the ability in general use even with the data of bipolar recording and scalp Laplacian; with additional channels in forward calculation, the EEG potentials at the missing channels can be recovered by the interpolation function of REST (Hu et al. 2019).

As the neural sources localization does not depend on the reference (Pascual-Marqui and Lehmann 1993), (10) should be theoretically efficacious in searching the sources as (2) that is impossible in practice. The sources **s** may be the actual or the equivalent sources that can generate the same scalp potential **φ**, based on the equivalent source principle (Dampney 1969; Yao 2000b; Yao and He 2003). To find the actual **s** by solving (10), it is difficult partly due to the nonlinear relations between **v**_*r*_ and the sources positions. The goal of REST is not to find the actual sources which one does not need to disentangle. One may take a closed distributed dipole layer with all actual sources inside as the equivalent sources (Yao 2000b; Yao and He 2003). Then (10) is a linear equation from the scalp data ${\mathbf{v}}_{r}$ to the strengths of the equivalent sources with fixed positions. Since the number of sources is usually much larger than the decayed rank in ${\mathbf{G}}_{r}$, (10) is an undetermined system. Thus, the pseudoinverse of ${\mathbf{G}}_{r}$ can be adopted to get the minimum norm solution to ${\mathbf{s}}$. Equation (10) also shows us that the sources ${\mathbf{s}}$ just play a role of bridge from ${\mathbf{v}}_{r}$ to **φ**. However, this bridge does lend the chance for REST from any unipolar reference recordings to **φ** at infinity (Yao 2001).

## Comparison of Unipolar References by Simulations

### Reconstruction of the Reference Signal

**Fig. 3:**
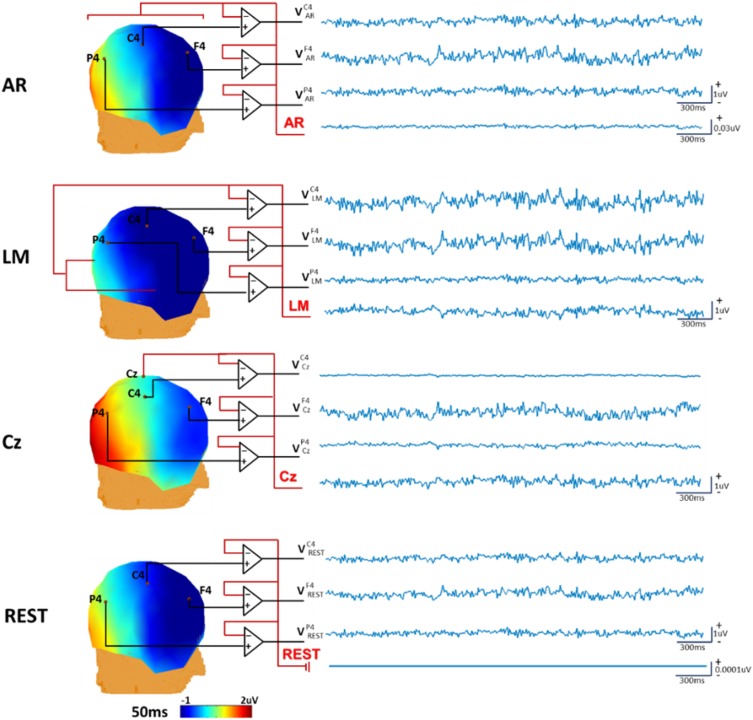
Simulated data illustrates the reference signals. With the same simulation procedure in Fig. 2, only two dipoles were used to simulate the EEG potentials and the sensor noise was not considered. The re-referenced scalp potentials and the topographies at 50 ms were displayed. The reference signals are displayed at the last trace of each panel

Figure 3 is the diagram of the unipolar references, such as AR, LM, Cz and REST. The simulation scheme is the same as that in Fig. 2. The referenced EEG and the reference signal traces (1–300 ms) are displayed. The signals of AR and LM are obtained from the average of potentials over all channels and two channels of mastoids, respectively. The head model in REST is built by FEM but the equivalent source space is consisted of 27921*3 discrete dipoles with x, y, z directions. The signal of REST is the difference between the forward recordings with infinity reference in Fig. 2 and the REST reconstructed recordings. The results showed the signals of LM and Cz are evident, and that of AR is small but nonnegligible, while REST almost recovers the actual zero potential. Recently, the AR signal in Fig. 3 was the yielded average oscillation (YAO) by REST and found as an electrophysiological signature of the resting-state fMRI global signal (Huang et al. 2018).

### Sensitivity to Errors in the Head Model

Theoretical advantage of REST is its use of the volume conductive model which practically depends on the factors: (a) the co-registration of electrodes with the scalp surface built from structural MRI T1 image; (b) neural sources modeling; (c) the head geometry model; (d) the conductivities of head tissues. One can only approximate the living human head consisted of the complex biological tissues and structures with many unknowns by numerical estimation, such as the geometry modeling by means of structural MRI T1 image under boundary condition and the isotropic/anisotropic conductivities of different tissues. No matter how fine the numerical head model is, it will still deviate from the truth in the sense of anatomy and physiologies. In addition, the neural sources can only be modeled with the assumption on the number and position of the actual sources. Furthermore, the electrode location deviation will introduce the error to the volume conduction model as well.

The preciseness of the volume conduction model is a common issue not only for REST but also for the electromagnetic source inverse solution. However, one may conjecture that the additional ‘forward’ step to the ‘inverse’ step, that is the estimation to the equivalent source in REST, makes REST probably more sensitive to the accuracy of head model than the other references such as AR.

**Fig. 4:**
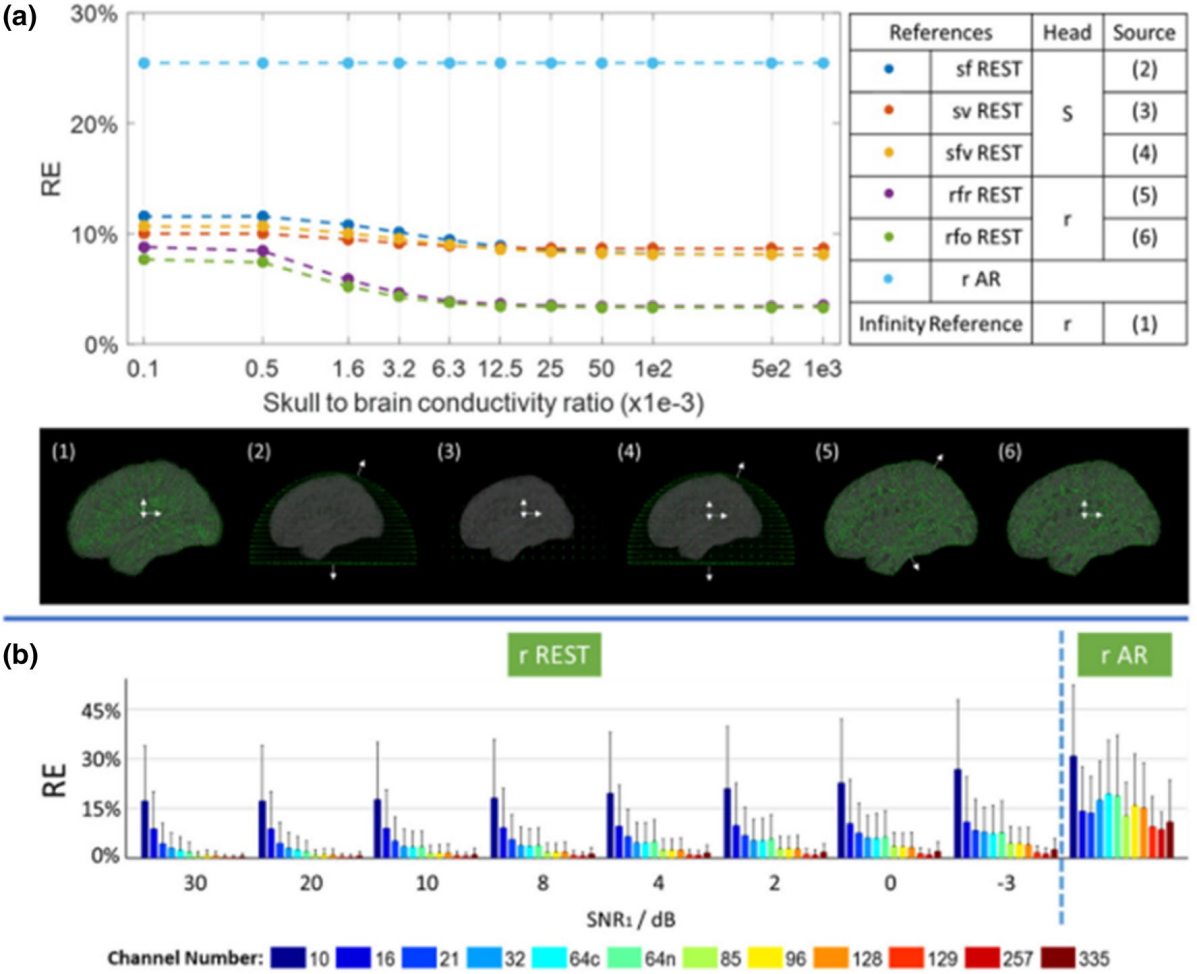
Simulated data tests the robustness of REST to errors in head models. **a** RE—the relative error; 1e2, 5e2, 1e3, and 1e-3 mean 100, 500, 1000, 0.001, respectively. **b** The numbers in the x-axis are the SNRs in dB; gray bars—the standard deviation of the REs over different sources. (Reproduced from Hu et al. 2018b)

To mitigate this concern, what (Hu et al. 2018b) tested is taking a very fine volume conduction model with infinity reference in generating EEG potentials, then reconstructing the potentials using REST where the volume conduction model is an alternative or the perturbated by injecting errors to that in the simulation.

(Hu et al. 2018b) investigated five alternatives shown in the Fig. 4a and perturbated volume conduction models by injecting the gaussian noise at different levels shown in the Fig. 4b. Specifically, the very fine volume conduction model in generating the EEG potentials is displayed in the Fig. 4a (1) and the one used in REST are illustrated as the Fig. 4a (2–6) with different source number and orientations. Using the prefixes to indicate the head shape and source configuration, the five alternatives are ‘sf’—homogenous Spherical head and cortical surFace dipoles with radial orientation, ‘sv’—homogenous Spherical head and brain Volume dipoles with orthogonal direction, ‘sfv’—homogenous Spherical head and cortical surFace dipoles together with Volume dipoles, ‘rfr’—Realistic head and cortical surFace dipoles with perpendicular (R) orientation, and ‘rfo’—Realistic head and cortical surFace dipoles with Orthogonal directions. Figure 4b shows perturbating the volume conduction model used in generating the EEG potentials with the gaussian noise at different levels as the one used in REST. The results demonstrate that REST is robust to reach the less potentials error than AR.

### Sensitivity to Neural Source Position

**Fig. 5:**
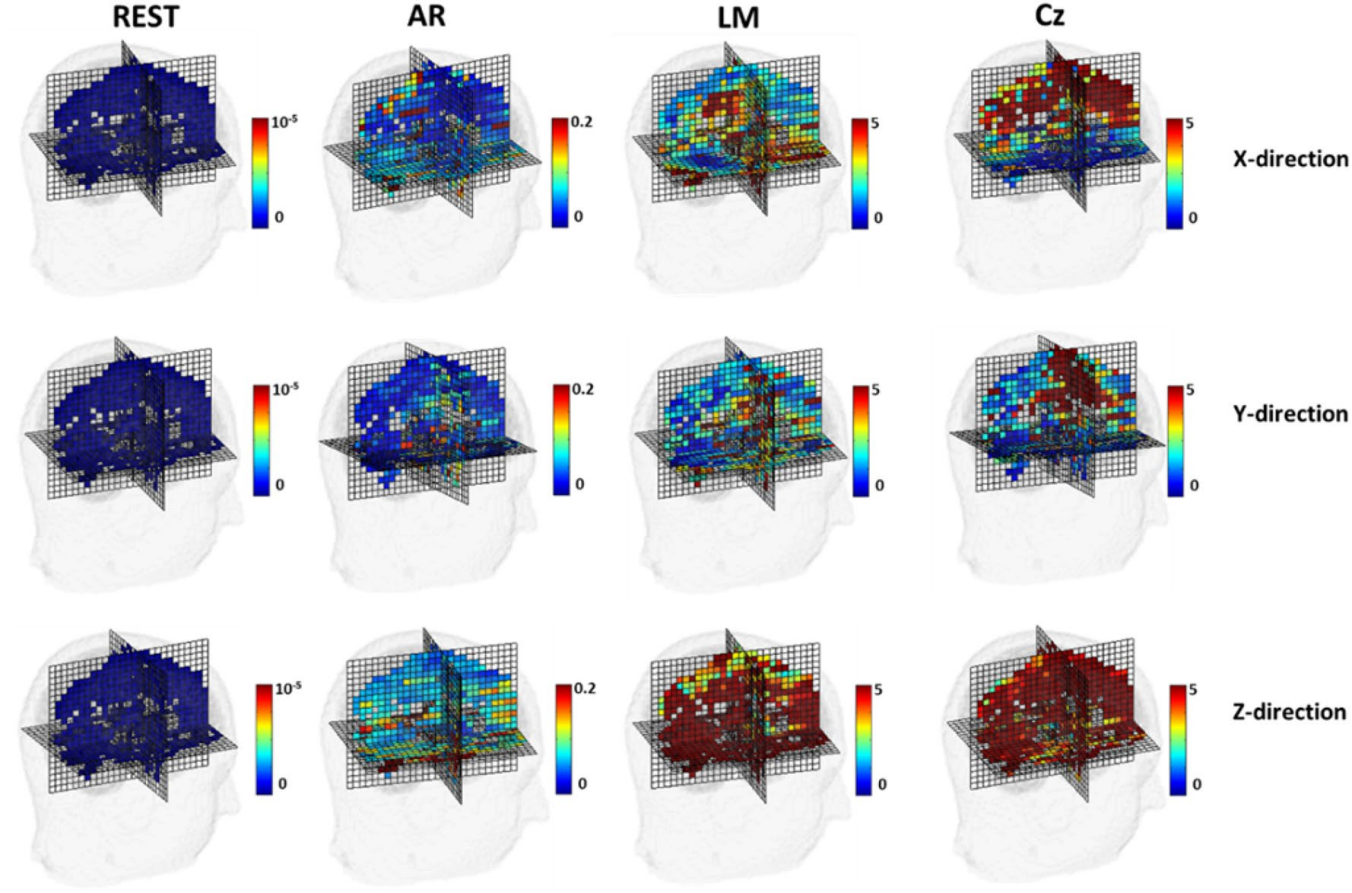
Simulated data with individual source shows the REs of potentials due to referencing. RE at each source is plotted at its position oriented to x-, y-, z- axis, respectively. The white voxels mean no sources

To test the sensitivity of unipolar references to the source position, simulation was conducted with each source repeatedly. Using the same volume conduction model in Fig. 2, the scalp potentials were generated by each of 3471*3 dipolar sources individually. For REST, we use the same head model built by FEM, but the equivalent sources were 27921*3 dipoles with x, y, z directions. Relative error (RE) for each dipole between the forward noisy free scalp potential with infinity reference and the potential with a reference is calculated and displayed at its location. Figure 5 is the display of the RE distribution of the potentials generated by each source with different references. Clearly, REST is always of the smallest errors contrast to the infinity reference potential, and AR is usually much better than LM and Cz. For AR, LM, Cz references, the errors depend on the dipole location and orientation. In addition to FEM based forward model, simulated results of potential errors before and after referencing were also investigated by using spherical head model and boundary element method (BEM) based realistic head model (Zhai and Yao 2004a; Hu et al. 2018b). Simulations with different forward models show the similar results.

### Sensitivity to the SNR and Head Model

There are two factors that might potentially affect AR or REST estimates of the potential at infinity. The first is the EEG signal to the sensor noise ratio (SNR) which will always affect the performance of inverse methods. Actual EEG measurement would not only record the physiological potentials but also unavoidably introduce the sensor noise. The second factor is the sensitivity of AR and REST to the underlying assumptions that lead to the estimators. As discussed before, AR conceptually depends on the measurement of potentials over a spherical head as well as the assumption of IID recordings. REST in turn utilizes the IID sources and a specific volume conduction model. The regularized versions of AR and REST termed as rAR and rREST (Hu et al. 2018c) therefore allow us for assessing these factors.

We carried out a simulation based on 89 individual realistic lead fields obtained from 89 subjects in the Cuban Human Mapping Project database (Hernandez-Gonzalez et al. 2011). Two patches of 150 dipolar sources with four order autoregressive model are used to produce the source time series; the individual lead fields are used to generate the potentials for each subject; the different SNRs were set as 20, 8, 4 and 2 dB. For rAR and rREST, the generalized cross validation (GCV) is used to select the denoising parameter. A usual direct measure to evaluate references is the relative error (RE) of referenced potentials against the simulated potentials at infinity. Four alternative head models were explored in (Hu et al. 2018c):

1. The usual lead field for REST based on a three-layer concentric spherical head model (SLF);
2. The realistic individual lead field calculated by FEM for each subject (ILF);
3. The averaged lead field over 89 subjects (ALF);
4. The sparse individual lead field with the known sources’ localizations (sILF).

**Fig. 6:**
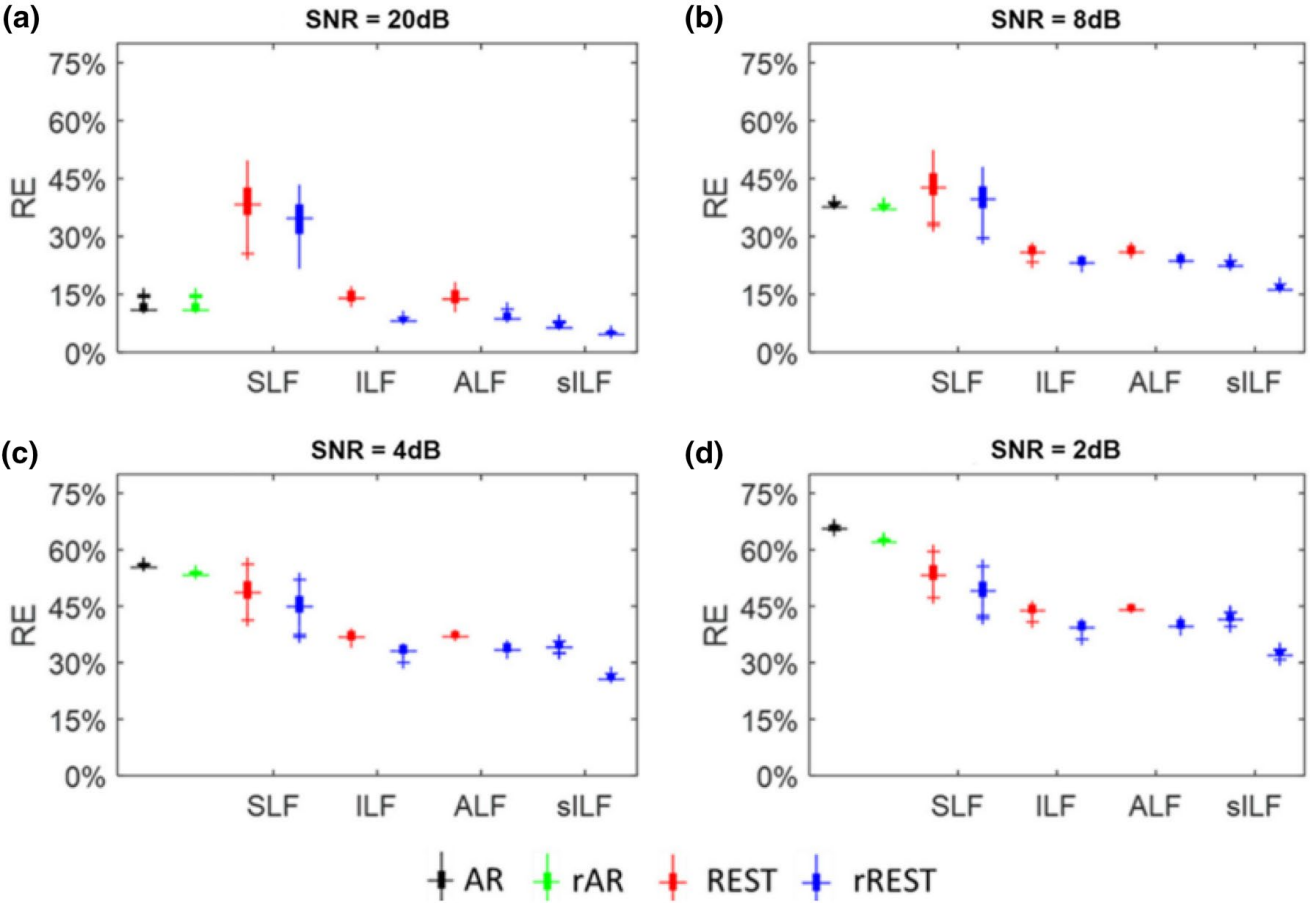
Simulated data tests the robustness of AR and REST on head model and sensor noise. *SLF* three-layer concentric spherical head model, *ILF* normalized individual head model, *ALF* averaged head model over all 89 subjects, *sILF* individual head model with sparse sources, *rAR* AR with denoising, *rREST* REST with denoising parameter selection and finer head model than SLF. The panels a–d are with different noisy levels (Reproduced from Hu et al. 2018c)

The results shown in the Fig. 6 are

- In high SNRs, the matching of head model used for REST and the one used for simulation becomes the main factor to affect the relative error. Therefore, REST with SLF are worse than AR with SNR = 20, 8 dB;
- With the SNR decreased, the impacts of noise overwhelm that of the matching of head models for REST. For any realistic SNRs = 4, 2 dB, any of REST models performs better than the AR. It is the denoising technique that greatly reduces the relative error of REST although using the SLF;
- rREST is more robust than rAR with the regularization technique in terms of denoising;
- rREST models achieve less relative error than REST; for sophisticated studies, better accuracy is achieved with the most accurate head and source models by rREST;
- Unless the real EEG recording is with extremely high SNR, REST with SLF can also be used without the expense of building the realistic head models for which the structural MRI is definitely needed;
- In general, the averaged lead field (ALF) over a population and the denoising technique of GCV should be used in the rREST practice.

## Impact of the Unipolar Reference on Real Data Analysis

Improper unipolar reference may introduce an unknown nonneutral value momently to all active channels as shown in Fig. 3, thus it definitely has distinct information criteria for reference model selection (Hu et al. 2018c) and different effects on waveform related parameters, such as amplitude, latency, spectra and their derived measures like coherence (Marzetti et al. 2007), network (Qin et al. 2010; Chella et al. 2016; Huang et al. 2017), bi-spectra (Chella et al. 2017) and statistical test (Tian and Yao 2013). Here examples related to information criterion, spectra, amplitude and latency of ERP are shown below.

### Evaluation of the References by Statistical Information Criteria

**Fig. 7:**
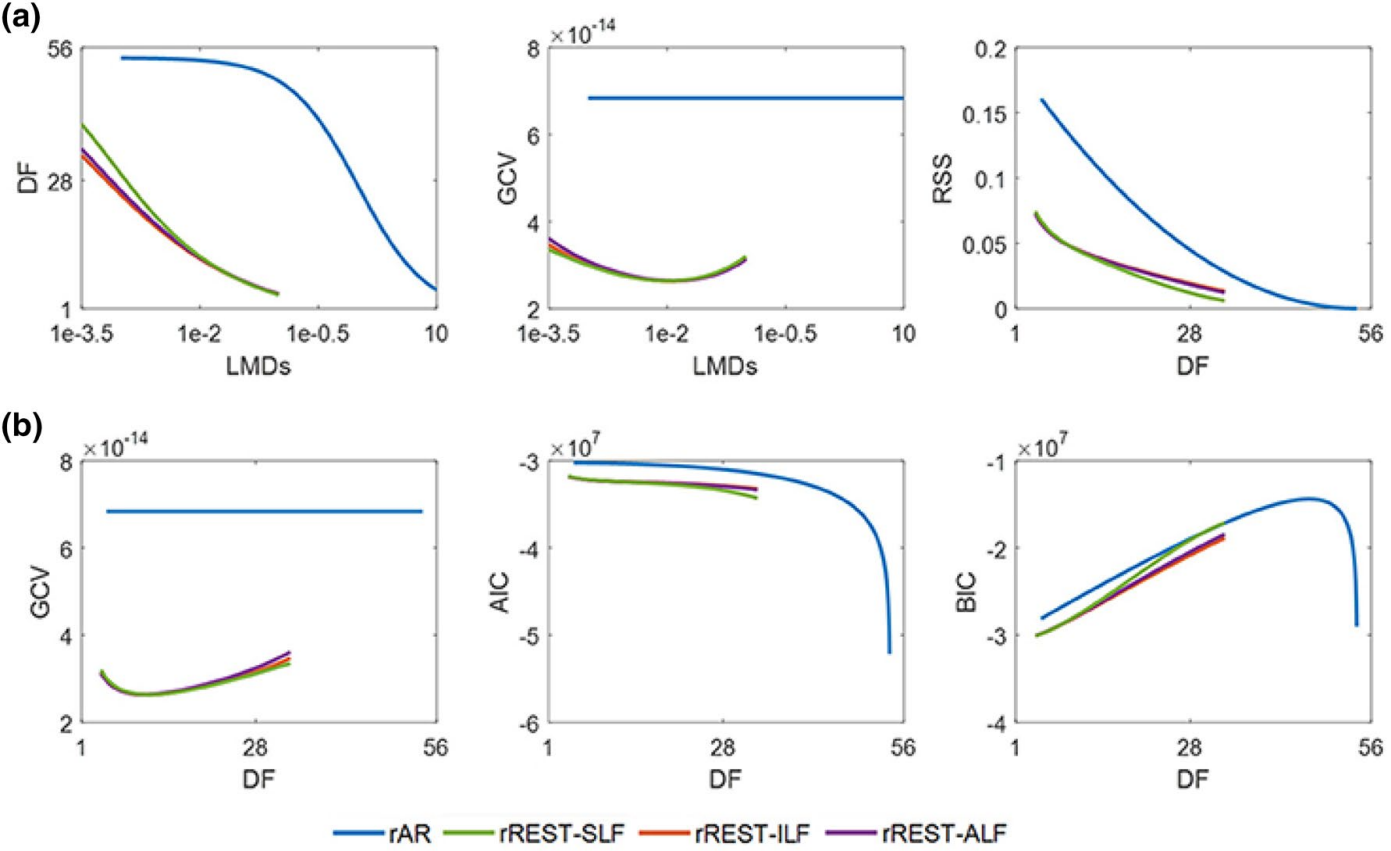
Recorded data derives the evaluation of references by the statistical information criteria. *DF* degree of freedom, *GCV* generalized cross validation, *RSS* the potentials residuals sum of square, *AIC* Akaike information criterion, *BIC* Bayesian information criterion, *LMDs* the denoising parameters, *SLF* spherical head model, *ILF* individual head models, *ALF* averaged head models over 89 subjects, *rAR* denoising average reference, *rREST* regularized reference electrode standardization technique. (Reproduced from Hu et al. 2018c)

In “Sensitivity to the SNR and Head Model” section, we reported the results of a statistical comparison of different references using simulated EEG data. However, it is much more important to evaluate the actual statistical adequacy of the different REST models against real data. This is carried out in (Hu et al. 2018c) by employing several statistical information criteria which balance the goodness of fit and model complexity (rAR, rREST-SLF, rREST-ILF, rREST-ALF). The results of this evaluation for the 89 subjects of the Cuban Human Brain Mapping database (Hernandez-Gonzalez et al. 2011) are shown in the Fig. 7, in which the curves are showing different statistical criteria, e.g. the generalized cross validation (GCV) versus different equivalent degree of freedom (DF) of AR and of the REST models. As is evident from the curves, any of the REST models achieves much smaller values for the statistical criterion than the AR except for the BIC of rREST with SLF when the DF is around 28. In statistical model selection, the model with smaller information criteria is preferred (Robert 2007; Konishi and Kitagawa 2008). And for all the practical purposes, the performances of all the rREST with realistic head models (ILF, ALF, sILF) are equivalent. The last plot in the panel b shows that when no denoising technique applied or the case of extremely high SNR (LMDs being around 1e-3.5 and DF being around 28 in the first plot of panel a), the BIC of rREST with SLF is coincided with the BIC curve of rAR, reinforcing our simulated result in the “Sensitivity to the SNR and Head Model” section that unless there is an extremely high SNR one can use the simplest SLF.

### On the Power Spectra of EEG

**Fig. 8:**
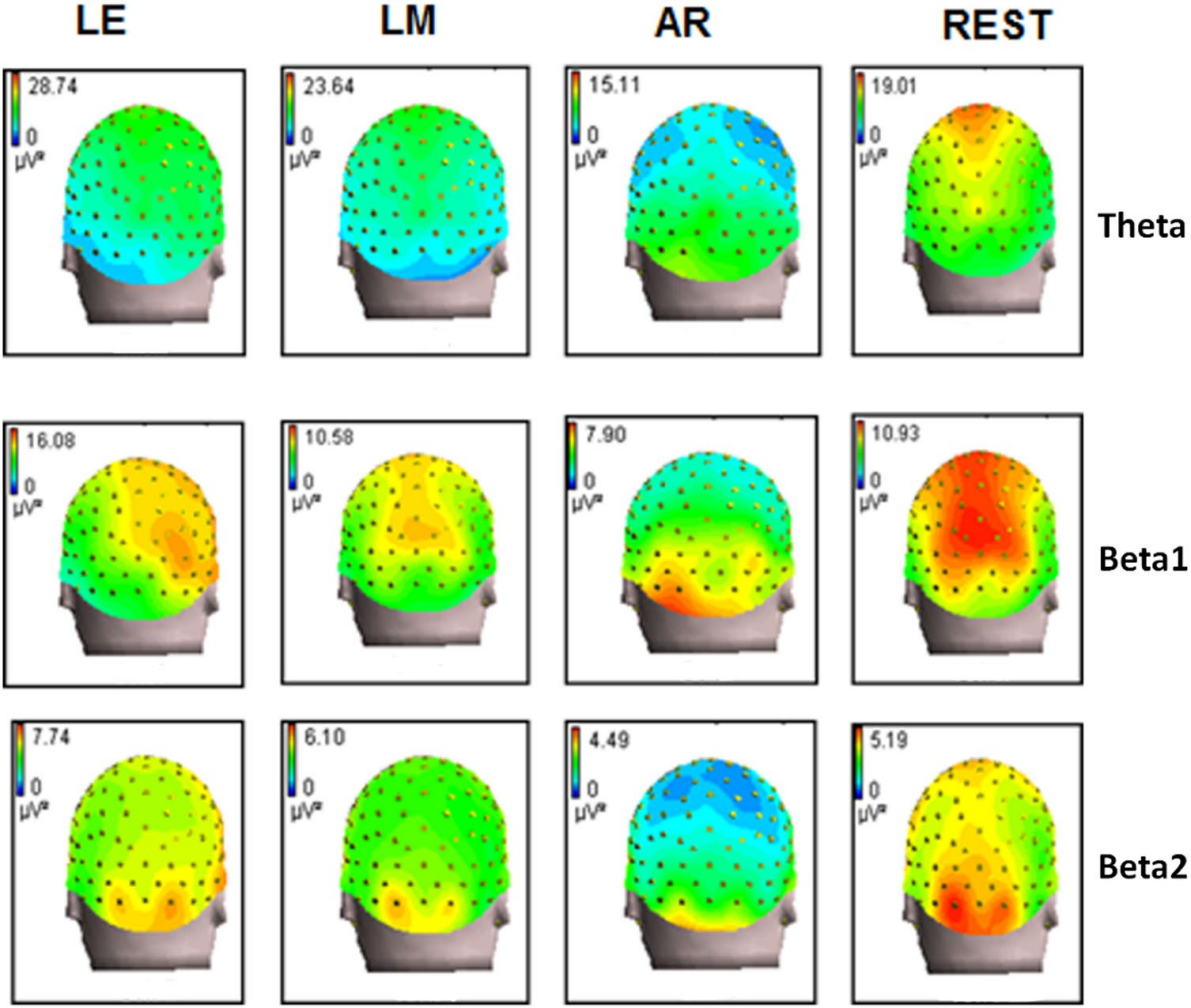
Recorded data derives the power spectra maps of EEG with different references. From left to right, the references are left ear (LE), LM, AR and REST. Compared to REST, the power spectra maps show shifts to the right, frontal and superficial positions with LE, to frontal and superficial positions with LM, and to a deeper position with AR. (The Alpha 1 and Alpha 2 maps were reported in Yao et al. 2005)

For the spontaneous EEG with a nonneutral reference signal mixed in other channels, the scalp power spectra map might be altered systematically. Figure 8 shows the results of theta, beta-1 and beta-2 using the same resting EEG data of 11 subjects with eyes open and consistent processing procedure as (Yao et al. 2005) where the results of alpha-1 and alpha-2 were reported. These results confirm that different references result in systematic changes in the distribution of EEG spectra power. It is therefore necessary to adopt a common prevalent reference and reduce the effect of such systematic shifts, allowing for the interpretation of the consistent field maps.

### On the Amplitude of the ERP

Waving sea level will change the height of a mountain contrast to the sea-level instantly but not alter the mountain shape. Analogously, a nonzero reference will change the amplitude of ERP component, but not alter the topographic distribution. Could the amplitude change have different interpretation?

**Fig. 9:**
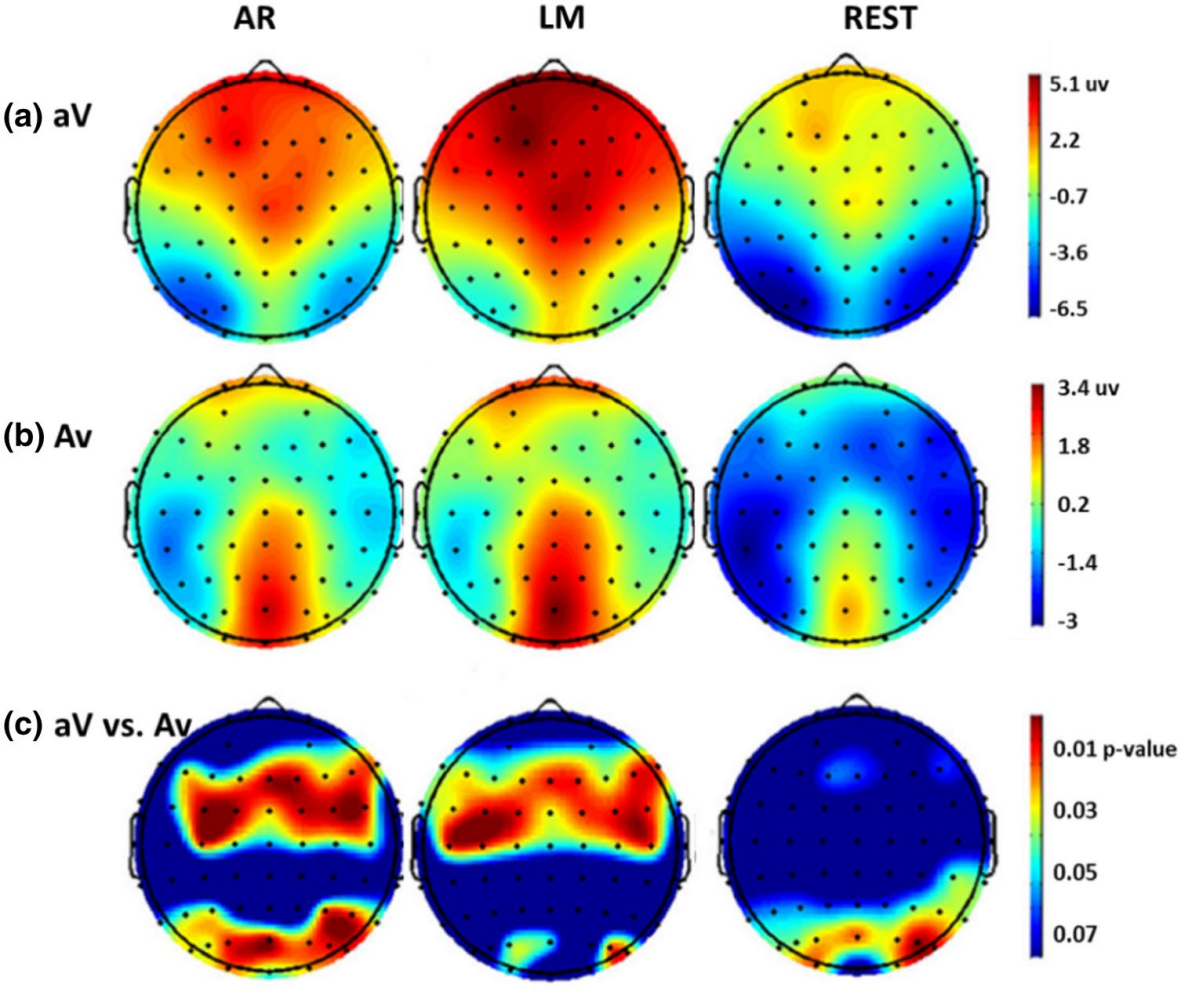
Recorded data derives the potential topographies and SPSM of N1 peaks (at 170–190 ms). **a** Potential topographies of attending V in an AV stimulus (aV), at 170–190 ms after the stimulus onset; **b** Potential topographies of attending A in an AV stimulus (Av), at 170–190 ms after the stimulus onset; and **c** SPSM (aV vs. Av) at 170–190 ms (Reproduced, with permission, from Tian and Yao 2013)

In psychological study, one ERP subtracting another ERP is a common strategy to get the different response in two stimulus cases. As the nonzero reference values of the two cases may not be the same, the difference of ERP will depend on the reference adopted. Here is an example using ERPs in an audiovisual (AV) stimulus (Tian and Yao 2013). Three references, AR, LM, and REST, were comparatively investigated via ERPs and statistical parametric scalp mappings (SPSM) that is the scalp distribution of the significant statistical difference between two conditions (Tian and Yao 2013). Specifically, for the N1 (170–190 ms), the SPSM results showed an anterior distribution for LM, a posterior distribution for REST, and both anterior and posterior distributions for AR (Fig. 9). In (Tian and Yao 2013), the result of REST is consistent with that by LORETA (low resolution electromagnetic tomography algorithm) (Pascual-Marqui et al. 1994). Such a distinct difference might mislead the interpretation of the underlying mechanism, and an actual amplitude information would be the starting point for any following studies.

### On the Latency of ERP

Referenced ERP is obtained by subtracting the reference signal from the active electrodes. If the reference signal is nonzero, the subtraction would distort the amplitude; if the reference signal has the delayed phase compared with the other active channels, it would affect the latency as well.

**Fig. 10:**
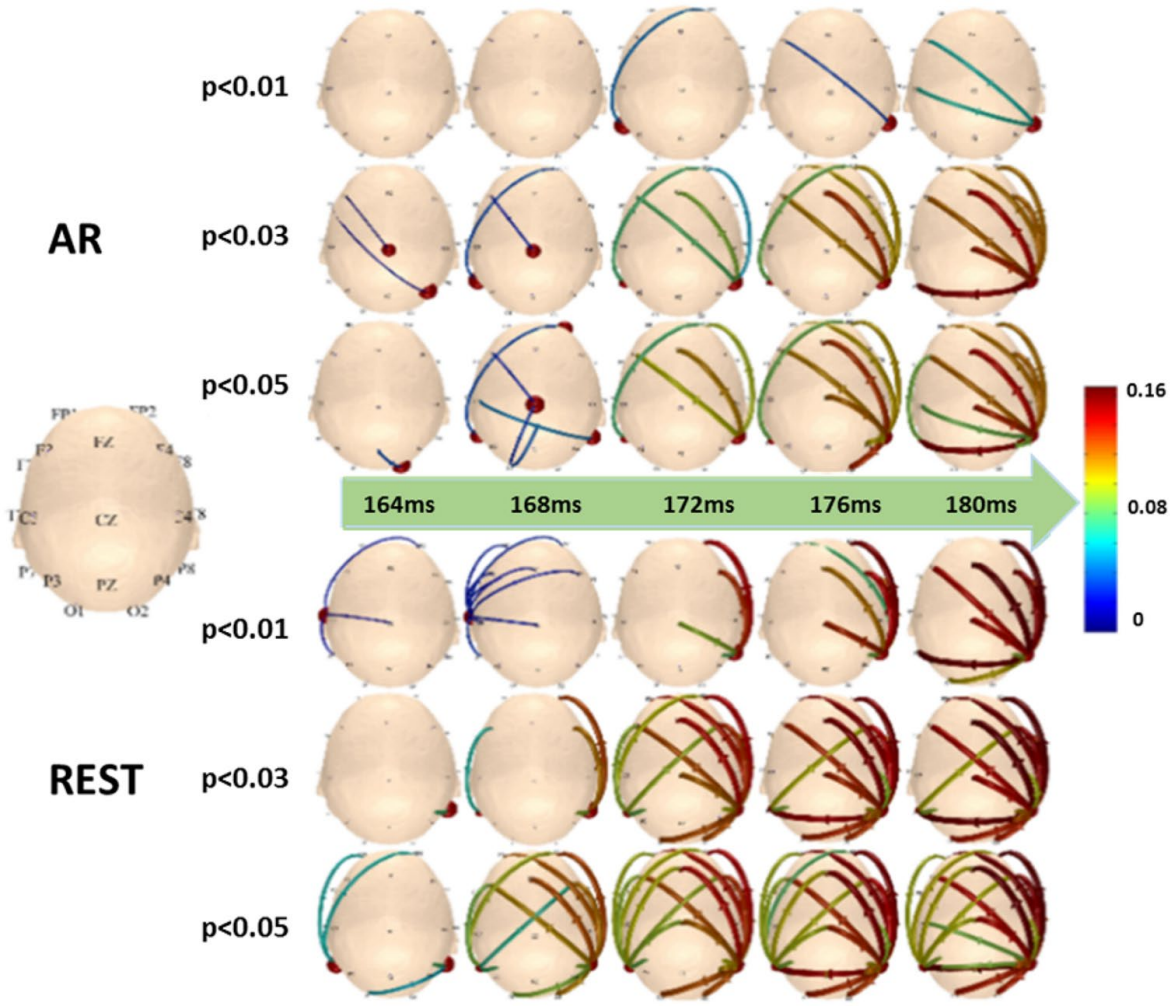
Recorded data derives the time-varying networks of N170. Hubs and connection mode change over time near 170 ms under the three different significance levels. (Reproduced with permission from Tian et al. 2017)

N170 is a negative ERP component appeared about 170 ms elicited by human face. The influence of the references on N170 was investigated using the scalp time-varying network method (Li et al. 2016). As the mastoids may be problematic for the N170 and other components, that are largest at lateral posterior electrode sites (Luck 2014). Two references, AR and REST, were comparatively investigated via the time-varying network processing of N170. Both AR and REST based networks show transfer function from the right P8 channel to the left. However, REST based result is more robust and earlier than AR based (Fig. 10). This phenomenon is further confirmed by a simulation study in (Tian et al. 2017). This means that reference is an important issue in precise investigation of the spatial–temporal dynamics of ERP, and REST based zero-reference would be the first step for the following explanation of various ERPs.

## Non-unipolar References

### Bipolar Recordings

**Fig. 11:**
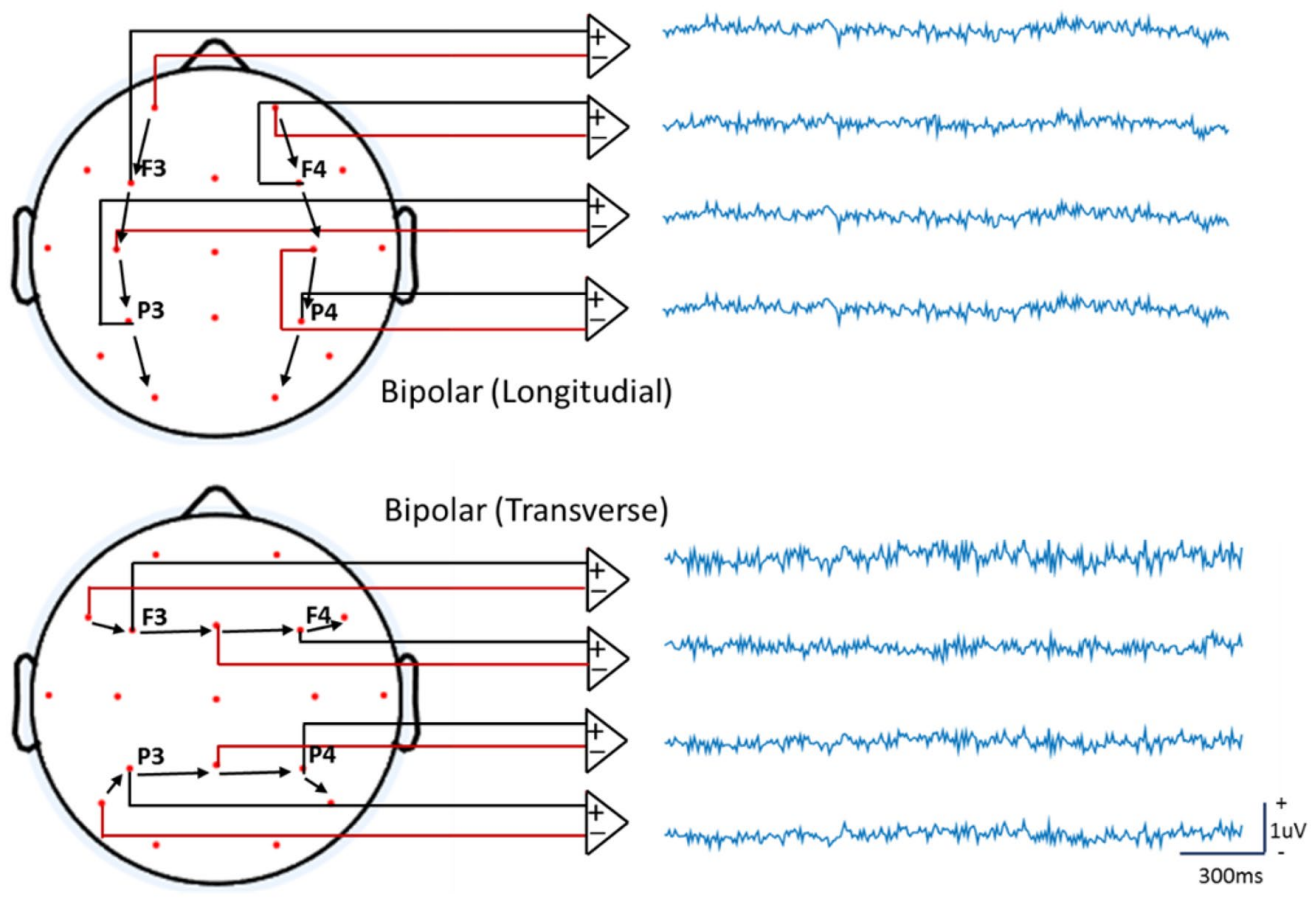
Bipolar recordings. With the same simulation procedure as in Fig. 2, it displays the bipolar longitudinal (upper) and transverse (bottom) recordings, respectively. The waveforms and polarities may be different at a moment for different montages. The waveforms here are distinct different from the unipolar recordings in Fig. 3

At the very beginning of EEG, Berger had only two electrodes for recordings. So he located the two electrodes within a part for the patients with partial skull missing and “front to back” mostly for the healthy subjects (Berger 1929; Stone and Hughes 2013). This is evolved as nowadays unipolar recording. Differently, the bipolar recordings is to estimate the potential differences between two adjacent electrodes. Any two electrodes may be subtracted to obtain one channel of bipolar recording. Currently, bipolar recordings are still widely used in clinical evaluation for epilepsy, where each electrode is typically referenced to an adjacent electrode. The bipolar montage may be in the longitudinal/anteroposterior direction, or the transverse/coronal direction (Niedermeyer and Da Silva 2005) illustrated in Fig. 11. In cognitive and affective neuroscience experiments, bipolar recordings is often used to measure the electrooculogram (EOG), that is the electrical potential caused by blinks and eye movements (Luck 2014). Mathematically, bipolar recording is a neighbor derivative, thus it is proportional to the local current density shown in the following:

15 $${\mathbf{c}} \approx {\mathbf{v}}_{r}^{(n + 1)} - {\mathbf{v}}_{r}^{(n)} \propto \frac{{\partial {\mathbf{v}}_{r} }}{\partial d}$$

16 $${\vec{\mathbf{j}}} \cdot {\vec{\mathbf{e}}}_{d} = \tau \vec{E} \cdot {\vec{\mathbf{e}}}_{d} = - \tau \nabla {\mathbf{v}}_{r} \cdot {\vec{\mathbf{e}}}_{d} { = - }\tau \frac{{\partial {\mathbf{v}}_{r} }}{\partial d} \propto {\mathbf{c}}$$

where vector ${\vec{\mathbf{j}}}$ is the current density, vector $\vec{E}$ is the electric field, $\tau$ is the conductivity of the scalp layer, ${\vec{\mathbf{e}}}_{d}$ is a unit vector from electrode $n$ to $n + 1$, $d$ is a unit distance scalar from electrode $n$ to $n + 1$, ${\mathbf{v}}_{r}^{(n)}$ is the potential at electrode $n$, ${\mathbf{c}}$ is the bipolar recording between electrode $n$ and $n + 1$. According to (15), the difference between two points detected by bipolar recording is actually an approximation of the 1^st^ order derivative of the potential. According to theory of electric field, it is a metric related to tangential current density over the scalp surface, not a potential at all, as illustrated by (16). Obviously, it depends on the montage. It is more sensitive to noise than to EEG signal, and less sensitive to signal from deep neural source because the derivative-like operation acts as a high pass filter. Bipolar recordings are mainly used in clinic to “enhance” focal activity (Niedermeyer and Da Silva 2005).

### Scalp Laplacian

(Hjorth 1975) proposed the use of a mathematical procedure for an estimation of brain generators of scalp EEG potentials. The procedure tried to estimate the orthogonal current through the skull entering (sink) or exiting (source) the scalp at each electrode site, so the result was originally called “orthogonal source derivation”. Scalp/Surface Laplacian (SL) is a discretization of the planar Laplacian operator, i.e. the difference between the potential at each electrode and the averaged potential of its nearest four neighbors.

In practice, SL can be estimated by a simple subtraction of a channel ${\mathbf{v}}_{r}^{(n,m)}$ from its four neighbors (Hjorth 1975).

17 $$i_{xy} = \frac{{\partial^{2} {\mathbf{v}}_{r}^{(n,m)} }}{{\partial x^{2} }} + \frac{{\partial^{2} {\mathbf{v}}_{r}^{(n,m)} }}{{\partial y^{2} }} \propto ({\mathbf{v}}_{r}^{(n + 1,m)} + {\mathbf{v}}_{r}^{(n - 1,m)} + {\mathbf{v}}_{r}^{(n,m + 1)} + {\mathbf{v}}_{r}^{(n,m - 1)} - 4{\mathbf{v}}_{r}^{(n,m)} )$$

Alternatively, we may also first fit the scalp discrete data to a continuous function, such as spherical harmonic function for spherical surface (Pascual-marqui et al. 1988; Perrin et al. 1989), a spline function for realistic head model (Babiloni et al. 2001), a radial-basis function (Yao 2002a; Zhai and Yao 2004b), then conduct a 2nd order analytical derivatives of the function.

The physical meaning of SL depends on the head model. (17) implicitly assuming the scalp as a plane, then combining with Eq. (1), SL is an estimate of the current source density (CSD) (Hjorth 1975; Yao 2002a). However, if the scalp layer of human head model as a cubic element, SL will be an estimate of local current density/flux (CD) through the skull into the scalp (Yao 2002a; Nunez and Srinivasan 2006). If the scalp layer as a more realistic spherical shell model, SL and local CD are related by a complex and nonlinear function of spatial frequency. But for practically low spatial frequencies, they are approximately linearly related, so one may consider SL as an approximate CD in practice (Yao 2002a). These indicates that the physical meaning of SL, CSD (Tenke and Kayser 2005) and CD (Giard et al. 2014) is undetermined but dependent on the head model assumed.

**Fig. 12:**
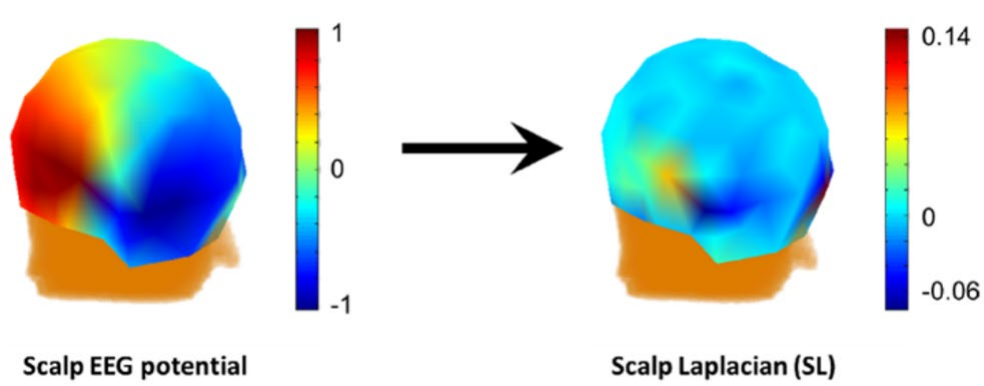
Scalp Laplacian. The neural activities are the same as in Fig. 2. Left: the scalp potential distribution at 100 ms; right: scalp Laplacian was calculated with the spherical spline method in Fieldtrip toolbox

Anyway, SL/CSD/CD is not potential in nature, free of the potential unipolar reference puzzle. As a different metric of the neural activities, approximately, the normal current (CD) passes through the skull into the scalp layer or the local CSD of a scalp point. SL may be used to illustrate local activities and called a high-resolution spatial imaging method (Fig. 12) (Yao 2000b). However, as a 2nd order derivative of the potential in (17), it is highly sensitive to the noise with wide spectra, and low sensitive to the deep sources. Actually, either direct measurement (Besio et al. 2006) or numeric calculation of SL is still a problem in debate. Depending on the head model shape, noise level and electrode density, various methods are developed, e.g. local numeric derivatives (Hjorth 1975), spherical harmonic Fourier expansion (Pascual-marqui et al. 1988), global spherical spline approach (Perrin et al. 1989) and moderate scale radial-basis function approach (Yao 2002b). Due to the pros and cons of SL, (Luck 2014) recommended to examine both the potential waveforms and the current density waveforms together.

## The Choice of Reference in Practice

Due to the different physics, each reference would be used under suitable and valuable situations. As noted from (Hu et al. 2019): “*The ‘no memory’ property of unipolar references means that one can re-reference the EEG/ERP recordings with different unipolar references but re-referencing won’t accumulate artifacts. Transforming from non-unipolar reference to unipolar reference will damage the dataset and it is no problem to transform the data within the unipolar references.*” When Laplacian is infeasible and bipolar is unacceptable, unipolar reference is a proper choice.

Table 1 provides a summary of the prevalent unipolar references and the frequently noted factors: electrode setup (density, coverage) and head model (shape and volume conduction). Apparently, online recording reference and offline LM are independent to the recording montage, and they are totally determined by the signals at the picked reference electrodes. Their main problem is the fact that the potential at the reference electrode is not constant as they are also generated by the dynamic sources inside the brain.

The AR and the REST are hoped to recover the infinity reference. Their accuracy depends on the assumption behind and the montage—the available channel information (Hu et al. 2018b). Their assumptions are based on the volume conduction model therefore affecting the feasibility of the method.

### Recording Reference (RR)

Recording reference is mainly adopted online before digital EEG era. One needs to pre-choose the reference point, such as nose, chin and ear etc. where is relatively inactive by guess. For example, to explore the neural mechanism of visual cognition, some researchers may assume the activities around the ears are weak, then an ear (mastoid) is taken as reference, and usually they only analyze the channels on the middle line such as Pz, Oz and Fz as they are a little far away from the ears. In current digital EEG era, if the available channels number is limited (< 10) or the coverage is partial to local region such as that in wearable EEG device, the online recording reference would be a compromising choice, especially when the offline re-referencing is infeasible.

### Linked Mastoids (LM)

As noted in (Luck 2014), “*the whole reference issue is a bit of a pain, but one nice thing is that you can easily change the reference offline, after the data have been recorded. And you can do this many time to see what your data look like with different references (which I highly recommend you do)*”. Offline unipolar re-references are the main options in current EEG studies. Among the three typical offline unipolar references, LM was the earliest for which a referable paper is (Gibbs et al. 1935). It was believed to be better than nose reference (Faux et al. 1990). However, LM was later criticized due to failing to localize the origin of the psychomotor seizure since the reference electrode linked to the ears distorted temporal activity (Feindel et al. 2009). In cognitive neuroscience study, LM is still one of the widely-used references. But, the papers using LM mainly study the channels at the middle line of the scalp as people are aware of the distortions near the two ears. Online LM recording reference is not recommended, as physically linking the wires from these two electrodes creates a zero-resistance electrical bridge as short-circuit between the two hemispheres, which may distort the distribution of voltage over the scalp and reduce hemispheric differences (Nunez and Srinivasan 2006; Luck 2014). Now, when should we use LM offline? The following notes should be considered: (1) the activities near the two ears are believed to weak, or possibly cancelled each other and the channels of interest are mainly the middle line electrodes; (2) the recording channels are limited (≤ 10) making it difficult to implement the REST, AR or Laplacian.

### Average Reference (AR)

Offline LM is not to approach zero potential but just because of the guess that the averaged potentials of the two ears is close to zero. It is a subjective empirical assumption without theoretical proof. In contrast, inspired from the Wilson common terminal reference in EKG, AR was reported in (Goldman 1950; Offner 1950); and there was a theoretical proof confirming the surface potential integral of a layered spherical sphere being zero (Bertrand et al. 1985). It was thus widely used in both EEG and ERP.

However, the integral may not be zero when a homogeneous and isotropic head is non-spherical (Yao 2017), and no one knows the situation for an inhomogeneous and anisotropic head. As shown by Table 1, the accuracy of AR depends on: whether a whole surface observation is feasible? Whether the electrode density is high enough to approximate the theoretical integral (Nunez 2010)? And whether the head is a homogeneous and isotropic spherical conductor (Bertrand et al. 1985; Yao 2017)? If all the answers are yes, it would be a golden standard (Nunez 2010).

However, the measurement cannot be on a whole head surface, the actual available surface is mainly the upper semi-head surface; the head shape is not spherical, homogeneous and isotropic but usually much more complex; the electrode arrays are usually not dense (Hu et al. 2018b). Comparatively, our recent work showed that the performance of AR has no close relation to the electrode density which is different from the usual understanding to AR based on its zero integral assumption, or say, coverage is a more important factor than the electrode density (Hu et al. 2018b). Therefore, AR cannot be a golden standard but an approximation.

So, when should we take AR in practice? Usually, AR may be an acceptable approximation if the subject head approximately closes to a sphere, and the montage is with good coverage, such as wider than a semi-head surface as the EGI system with enough density such as > 128 channels (Hu et al. 2018b). In general, we do not recommend using AR if the REST is available. Besides, in current digital EEG era, online AR is not recommended for the same reason as the LM. The additional limitation of AR is that one has to be sure the EEG data at hand is with unipolar references before applying AR (Hu et al. 2018a, 2019).

### Reference Electrode Standardization Technique (REST)

As confirmed, with the physical fact that all physiological scalp signals at both active electrodes and reference electrode are generated by the same brain sources, REST (Yao 2001) performs much better in recovering the actual potential on the scalp surface with the approximated infinity reference. In general, the accuracy of REST depends on the equivalence of the reconstructed equivalent sources and the unknown actual sources in generating the scalp potential, and it can be applied to any a complex head model. However, the lead fields in (12) involve the three factors in Table 1. Thus, the accuracy of REST may be improved with a wider coverage, denser observation and more realistic head model. REST would be a good choice for such a case: the electrode montage with a nice coverage that is at least the upper hemi-head surface, necessary electrode density (≥ 16), acceptable approximate head model (the concentric three-sphere head model or MRI image based realistic head model). Generally, REST would be the best for most cognitive studies and clinic EEG problem, which were repeatedly confirmed by a series of simulation studies (Zhai and Yao 2004a; Marzetti et al. 2007; Qin et al. 2010; Liu et al. 2015; Chella et al. 2016). Its rationality in processing various real data was also proven step by step (Yao et al. 2005; Bonfiglio et al. 2013; Tian and Yao 2013; Xu et al. 2014; Kugiumtzis and Kimiskidis 2015; Chella et al. 2016; Mumtaz and Malik 2018).

Two prominent advantages of REST are that (1) it adapts to the EEG data with unipolar, bipolar recordings and Laplacian transformed, whereas the strict prerequisite before applying AR is that the EEG reference needs being unipolar (Hu et al. 2018a, 2019). (2) with the additional channels in forward calculation, the EEG potentials at the missing channels rejected as bad channels can be recovered with the interpolation function of REST (Hu et al. 2019). Besides, one may worry about the possible limitations of REST: (1) sensor noise problem. Note the difference in (3) and (12). The model deducing REST is based on a noise free model. (2) the inaccurate head model may affect the robustness of REST. To address these two problems, we have introduced the generalized cross validation as the criterion to select the denoising parameter and proposed an averaged lead field over a population in the rREST practice (Hu et al. 2018c). In addition, the evaluations of REST were still limited to the layered homogeneous and isotropic head model such as the concentric three-sphere head model or three-layer realistic head model. Further updated head model accounting of anisotropic properties of skull and white matter will be valuable for its application.

### Non-unipolar References

Bipolar reference recordings are not the way to get the actual potential but show local surface potential variance of underlying neural activities as the 1st derivative of potentials. So if the actual potential is not interested, or the channels are insufficient (< 10) to apply REST (Hu et al. 2018b), and the main concern is local activities instead of the whole scene, then bipolar reference recordings may be acceptable, such as in neurological clinic where interictal epileptic spike or local abnormal electric current is interested (Reilly 2005). However, for cognitive and psychological studies, such a reference montage sounds never used. The distinct advantage of bipolar reference recording is free of the influence from electrode number and density. As one of the reviewers noted: “*Bipolar recordings are generally of very limited value when electrode separation is large. However, with small separations bipolar recordings provide estimates of the tangential electric field halfway between the electrodes. This approach has been used effectively to estimate the propagation speed of traveling waves of electric field across the scalp for both resting EEG and evoked potentials (independent of reference or head model)*”.

Laplacian montage is often recommended due to its reference-free nature and relatively higher spatial resolution. However, Laplacian is not a physical measure but the 2nd order derivative of the scalp potential (Lai and Yao 2009). Dense electrodes array (> 64) and high SNR are necessary to get valuable estimation over the whole scalp surface. In addition, Laplacian is more sensitive to shallow local source than to distributed deep source, and its estimates at the boundary channels are usually unreliable (Yao 2002a; Zhai and Yao 2004b). Thus, if the interested activities are located deeply or distributed and the concerned channels are close to the boundary channels, cautions should be taken when using Laplacian. Otherwise, it might be an accredited choice to get reference-free. Certainly, if online direct measurement of Laplacian by tripolar electrode approach is realized easily in the future, Laplacian may be used specifically for some points interested on the scalp surface even in wearable EEG system (Besio et al. 2004, 2006). As one of the reviewers noted: “*Laplacian should not generally replace the reference potential, rather it provides estimates of smaller scale source regions, thereby yielding additional and complementary information. The issue of noise depends very much on application. For example, the resting state alpha band consists of multiple source regions of different sizes and locations. A large local Laplacian, even if its magnitude is somewhat inaccurate, can indicate the presence of local sources within a much larger synchronous region. Furthermore, Fourier transform methods (including coherence) involve time averages over hundreds or thousands of time points, expected to substantially reduce noise errors*”.

## Summary

Many studies have shown that nonzero reference has distinct effects on waveform and related parameters, such as information criteria, amplitude, latency, power, phase, and further derived parameters e.g. coherence, correlation, network, symmetry, covariance and statistic test. A prevalent neutral unipolar reference is fundamentally important to comparison among different labs and the data collected and stored with different references over time.

The reference problem is a special issue for potential difference over reference electrode and the active electrode which would commonly record the filtered neural electric current from the same source by volume conduction. The problem of volume conduction cannot be perfectly solved in EEG/ERP recordings as the observed multichannel recording is rank-deficient (Hu et al. 2019), namely, the lost signal of the reference electrode cannot be recovered from itself, thus the information content of the offline LM or AR based recordings is the same as that of the online unipolar reference recordings such as Cz, Pz. Differently, REST tackles the problem by realizing the nature reason of volume conduction. All the known simulation studies confirmed that REST (rREST) is the best to approach the ideal unipolar infinity reference with golden standard data as the ground true, and it was recommended by the International Federation of Clinical Neurophysiology (IFCN) Guidelines (Babiloni et al. 2019) and the ”Best Practices in Data Analysis and Sharing in Neuroimaging using MEEG” of OHBM(https://cobidasmeeg.wordpress.com/). Now, a MATLAB toolbox including EEGLAB Plugin is listed at http://www.neuro.uestc.edu.cn/rest/Down.html (Dong et al. 2017), making it convenient to employ REST to remove the barrier to the actual data. Moreover, REST is now integrated in BEAPP (https://github.com/lcnbeapp/beapp), and will be integrated into EEGLAB software. As well, the codes of rREST are publicly available at https://github.com/ShiangHu/Unified-EEG-reference-rREST for the later update of the REST toolbox and constructing your own referencing protocols as MATLAB scripts which will be helpful in the batch processing of group level studies.

Meanwhile, non-unipolar reference such as bipolar reference recordings and Laplacian may be alternatives for clinic practice and shallow sources focused situations, respectively. As non-potential but the derivatives of the potential, they are far away from reference problem.
